# Molecular cloning, expression and the adjuvant effects of interleukin-8 of channel catfish (*Ictalurus Punctatus*) against *Streptococcus iniae*

**DOI:** 10.1038/srep29310

**Published:** 2016-07-04

**Authors:** Erlong Wang, Jun Wang, Bo Long, Kaiyu Wang, Yang He, Qian Yang, Defang Chen, Yi Geng, Xiaoli Huang, Ping Ouyang, Weimin Lai

**Affiliations:** 1 Department of Basic Veterinary, College of Veterinary Medicine, Sichuan Agricultural University, Chengdu 611130, Sichuan, China; 2 Key Laboratory of Animal Disease and Human Health of Sichuan Province, Sichuan Agricultural University, Chengdu 611130, Sichuan, China; 3 Department of Aquaculture, College of Animal Science and Technology, Sichuan Agricultural University, Chengdu 611130, Sichuan, China

## Abstract

Interleukin-8 (IL-8) as an important cytokine involving in inflammatory and immune response, has been studied as effective adjuvants for vaccines in mammals. However, there are fewer reports about the characterization and adjuvant effects of IL-8 in fish. In this study, cloning and sequence analysis of IL-8 coding region of channel catfish (*Ictalurus punctatus*) were conducted, mature IL-8(rtIL-8) was expressed and evaluated for its adjuvant effects on the immunoprotection of subunit vaccine encoding α-enolase (rENO) of *St*reptococcus *iniae* from several aspects in channel catfish. The results showed co-vaccination of rENO with rtIL-8 enhanced immune responses including humoral and cellular immunity, with higher relative percent survival(RPS,71.4%) compared with the moderate RPS of rENO alone(50%) against *S. iniae* infection at 4 week post vaccination. While rtIL-8 failed to maintain long-lasting immune protection, only with RPS of 26.67% in rENO + rtIL-8-vaccinated fish compared with that of rENO alone(20%) at 8 week, signifying that IL-8 hold promise for use as potential immunopotentiator in vaccines against bacterial infections in fish, whereas it is insufficient to extend the immunoprotection for long time, and further studies are required to understand the mechanisms of IL-8 used as an adjuvant and seek for more effective way to strengthen the adjuvanticity of IL-8.

Aquaculture known as one of the most vibrant sector of the global food system has made a significant contribution in the production of protein-rich food for human consumption^1^. However, aquaculture practices encounter many challenges in recent decades, and one of the most devastating problems is disease outbreaks caused by microbial pathogens. Channel catfish (*Ictalurus punctatus*) as the most extensively cultured food-fish species especially in the United States and China, suffers from widespread disease outbreaks due to various bacterial pathogens^2^,^3^. *Streptococcus iniae*, a Gram-positive bacterium and one of the β-hemolytic Streptococcus species, has caused serious damage to fish farming in recent years^4^,^5^,^6^,^7^, thereby becoming an important fish pathogen with a broad host range, including Asian seabass^8^, Nile tilapia^9^, zebrafish^10^, Japanese flounder^11^, rainbow trout^12^ and channel catfish^13^. In addition to fish, *S. iniae* is also known to be an opportunistic human pathogen and causes diseases in human^14^,^15^,^16^.

Traditionally, controlling of *S. iniae* infection in aquaculture relies mainly on the use of antibiotics and antimicrobial compounds. However, due to the indiscriminate use of antibiotics, several problems have arisen such as the development of resistance against antibiotics and safety concerns associated with antibiotic residues. Fortunately, vaccine has been suggested as an effective method and a green intervention to prevent *S. iniae* infection. Compared with inactivated and/or attenuated live vaccines, subunit vaccines composed of conserved proteins are safer and more serotype-independent^17^. Many *S. iniae*–derived proteins were identified as immunogenic^18^, and α-enolase (ENO) as one of the surface-exposed and highly conserved proteins between Streptococcus spp^19^, had been reported to be used as an efficient protective antigen candidate against *S. iniae* infection in mice according our previous study^20^. It was also confirmed that recombinant ENO (rENO) induced cross-protective immunity against *S. iniae* and *Streptococcus parauberi* in zebrafish^10^. However, poor immunogenicity has been the main constraint to subunit vaccine development. Thus, the use of adjuvants in subunit vaccines is exceedingly necessary and important.

Cytokines are a family of low molecular weight and secretory proteins which involved in various pro-inflammatory functions against invasive pathogens including bacteria, virus or parasite. Chemokines as a kind of cytokines are among the adjuvants more widely used for vaccination against various pathogens in mammals^21^,^22^,^23^. To date, many cytokines and chemokines have been found in bony fish, and their functions and signaling are being explored with great progress^24^,^25^,^26^. Moreover, several cytokines have already been shown to be efficient adjuvants which are crucial for vaccine effectiveness to obtain the appropriate immune response and ensure a protective outcome in fish^24^,^27^,^28^. Interleukin-8 (IL-8) is one of the first CXC chemokines to be discovered in fish^29^. Some studies have focused on the potential use of IL-8 as an adjuvant for genetic engineering vaccine in mammals, while in fish IL-8 has just been cloned and characterized in few species including lamprey^30^, Japanese flounder^29^, rainbow trout^31^, and channel catfish just with a report of an IL-8 like gene^32^, and an understanding of the biological role of IL-8 has not been achieved in any of these species.

In the current study, we cloned the IL-8 coding sequence of channel catfish, selected the truncate IL-8 (tIL-8, mature IL-8 regions) to express based on the sequence analysis, and evaluated its adjuvant effects on the protective immunity of a subunit vaccine encoding α-enolase (rENO) of *St*reptococcus *iniae* from several aspects including lysozyme activity, alternative haemolytic complement activity (ACH50), antibody titers, the expression of immune-related genes and relative percent survival (RPS) in channel catfish.

## Results

### Cloning, sequence and phylogenetic analyses of IL-8 coding regions

**Figure 1:**
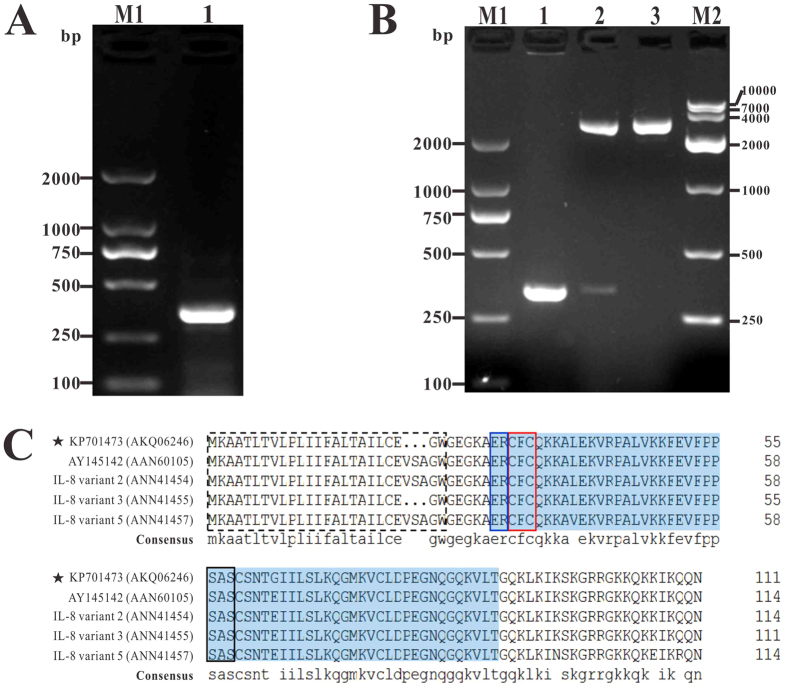
PCR amplification of IL-8 gene, identification of recombinant plasmid pMD19-T-IL-8 and sequence analysis of IL-8 amino acid sequence. (**A**) PCR amplification of IL-8 gene. M1: DNA marker (DL2000); lane 1: PCR product of IL-8 gene (336 bp). (**B**) Identification of recombinant plasmid pMD19-T- IL-8 with PCR and enzyme digestion. M1: DNA marker (DL2000); lane 1: PCR product of plasmid pMD19-T-IL-8; lane 2: digestion of plasmid with EcoR I and Xho I; lane 3: digestion of plasmid with XhoI; M2: DNA marker (DL10000). (**C**) Amino acid sequence analysis of IL-8 sequence with other channel catfish IL-8 s. Dotted box presented signal peptides, shadow regions presented the conserved Chemokine_CXC domain. The ELR motif, CXC motif and GPH motif were indicated by blue, red and black box, respectively.

The full-length of IL-8 coding sequence of channel catfish was 336 bp (GenBank No. KP701473) (Fig. 1A), which contained a complete open reading frame (ORF) and encoded a 111 a.a. with the predicted molecular weight of 12.20 kDa and the theoretical pI of 10.08. The a.a. sequence was predicted to contain a 23 a.a. signal peptide (Fig. 1C). Thus, the mature peptide of channel catfish IL-8 (tIL-8) was speculated to consist of 88 a.a. There was a Chemokine_CXC domain which was conserved in Chemokine superfamily locating at N-terminal of 30 ~ 88 a.a residuals of the IL-8. Three consensus motifs (ELR motif, CXC motif and GPH motif) existing in Chemokine_CXC domain were also found in channel catfish IL-8 at the N-terminal of 30 ~ 31, 32 ~ 34 and 56 ~ 58 a.a. residuals, respectively (Fig. 1C).

**Figure 2:**
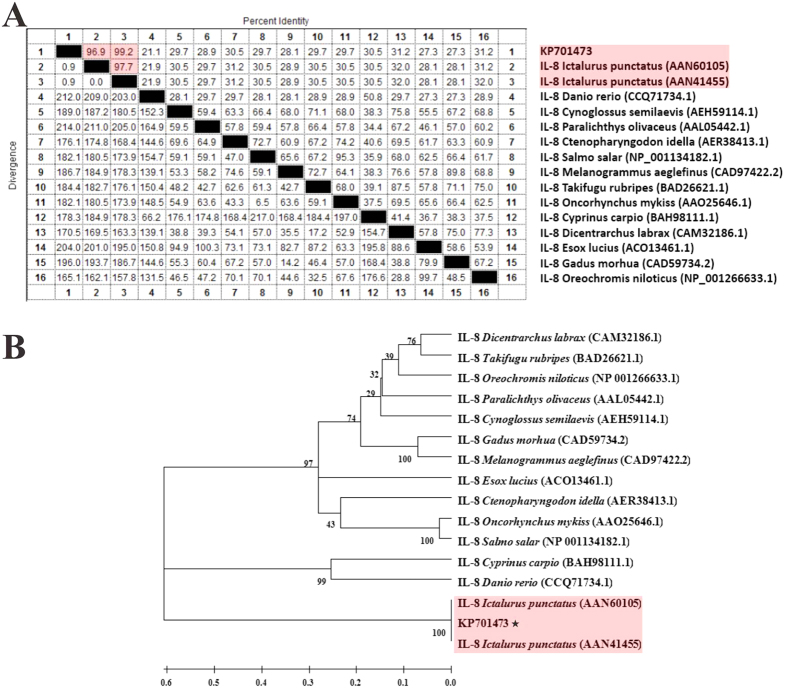
Multiple sequences alignment and phylogenetic tree of IL-8 amino acid sequences with 15 different fish IL-8s. (**A**) Multiple sequences alignment of IL-8 a.a. sequences. (**B**) Phylogenetic tree of IL-8 a.a. sequences. The numbers at each branch represent the bootstrap values obtained with 1000 replicates. “KP701473” represents the a.a. sequence of channel catfish IL-8 in this study.

The a.a. sequence identity between channel catfish IL-8 and 15 different fish IL-8s were calculated using Clustal W and MegAlign, which suggested that IL-8 in this paper shared high identity of 96.9 ~ 99.2% to channel catfish IL-8 (AAN60105 and AAN41455) with only 1 a.a. residual difference from AAN41455, and low identity ranging from 21.1 to 31.2% compared with other fish species (Fig. 2A). The phylogenetic tree constructed with Mega 5.1 showed that IL-8 in this study was clustered together with channel catfish IL-8 (AAN60105 and AAN41455) displaying the close relationship between them, with 100 bootstrap support (Fig. 2B), and a distant relationship existed between channel catfish IL-8 with other fish species.

### Molecular cloning, expression and purification of rtIL-8

**Figure 3:**
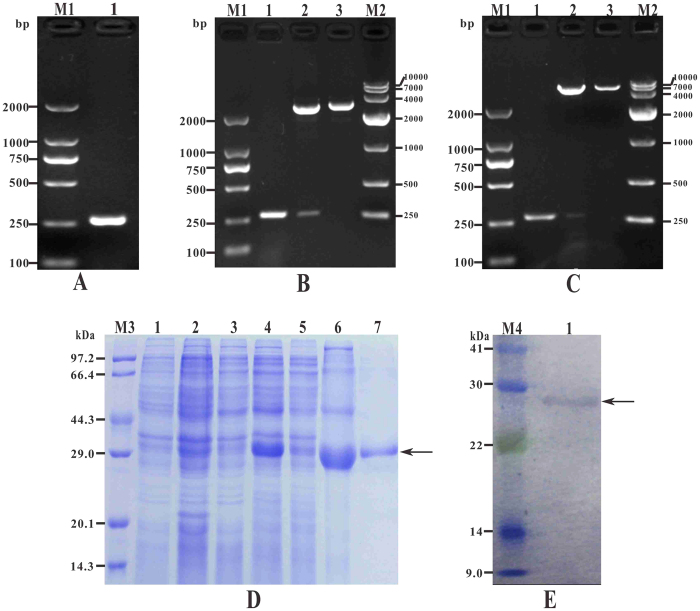
Molecular cloning, expression, purification and western blot analysis of rtIL-8. (**A**) M1: DNA Marker (DL2000); lane 1: PCR product of tIL-8 gene with 264 bp. (**B**) M1: DNA Marker (DL2000); lane 1: PCR product of recombinant plasmid T-tIL-8; lane 2: digestion of T-tIL-8 with EcoR I and Xho I; lane 3: digestion of T-tIL-8 with Xho I; M2: DNA Marker (DL10000). (**C**) M1: DNA Marker (DL2000); lane 1: PCR product of plasmid P-tIL-8; lane 2: digestion of P-tIL-8 with EcoR I and Xho I; lane 3: digestion of P-tIL-8 with Xho I; M2: DNA Marker (DL10000). (**D**) M3: protein marker; lane 1 ~ 6: uninduced BL21(pET32a), induced BL21 (pET32a), uninduced BL21 (P-tIL-8), induced BL21(P-tIL-8), induced BL21(P-tIL-8) supernatant, induced BL21(P-tIL-8) sediment, lane 7: purification of recombinant rtIL-8. (**E**) M4: protein marker; lane 1: Specific binding between recombinant protein rtIL-8 and rabbit anti-6 × Histidine antibody.

To determine the role of mature peptide of channel catfish IL-8 in the immune regulation, tIL-8 gene (not containing signal peptide) was successfully amplified with PCR and cloned into pMD19-T (Fig. 3A,B), followed by expectedly expressed with vector pET32a (Fig. 3C) in induced BL21(P-tIL-8) sediment and purified by Ni-NTA metal affinity chromatography. Single bands of about 29 kDa of recombinant proteins rtIL-8 were observed after overexpression and purification with SDS-PAGE (Fig. 3D), which was close to the predicted molecular weight. The protein concentration of the rtIL-8 solution was determined as 2.4 mg/ml. Western blot analysis showed that the 29 kDa band of recombinant proteins rtIL-8 reacted specifically with the rabbit anti-6-Histidine antiserum (Fig. 3E).

### Immunological analysis

#### Serum lysozyme activity

**Figure 4:**
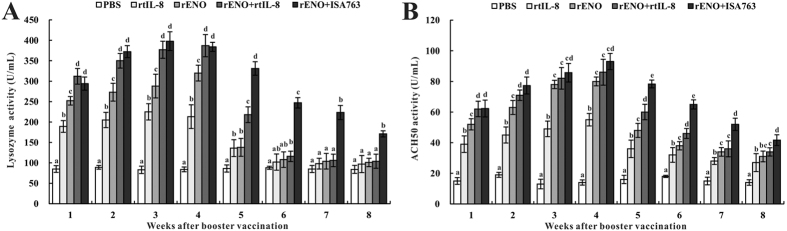
Serum lysozyme activity (**A**) and ACH50 activity (**B**) of vaccinated fish. Channel catfish were vaccinated twice at 2-week intervals, with PBS (control), rtIL-8, rENO, rENO + rtIL-8 and rENO + ISA763. Sera were collected from the fish at 1 ~ 8 week s.v. Data are presented as means ± SD (n = 5). Different letters above a bar denote significant difference (*P* < 0.05).

The serum lysozyme activity of vaccinated fish was assessed by a turbidimetric assay from 1 to 8 week post-secondary vaccination (s.v.) (Fig. 4A). In general, the four vaccinated groups (rtIL-8, rENO, rENO + rtIL-8 and rENO + ISA763) revealed significantly higher levels of lysozyme activity compared with those of control group from 1 to 5 week s.v. with the peak value occurring at 3-week, 4-week, 4-week and 3-week s.v. respectively, and the highest lysozyme activity located in rENO + ISA763 group at 3-week s.v. Furthermore, the lysozyme activity of rENO groups were remarkably higher than that in rtIL-8 group from 1 to 4 week s.v., and the combination of rENO with rtIL-8 enhanced more markedly the action of lysozyme activity than did rENO from 1 to 5 week s.v. Although the difference between rENO + rtIL-8 and rENO + ISA763 were not significant in 1 ~ 4 week s.v., it was significant from 5 to 8 week s.v.

#### Serum ACH50 activity

During all experimental time points, the serum ACH50 activity of four vaccinated groups increased significantly compared with control group (Fig. 4B), and the highest ACH50 activity were all observed at 4-week s.v. in four vaccinated groups. rENO induced remarkably higher ACH50 activity than rtIL-8 from 1 to 7 week s.v., while the ACH50 activity of rENO + rtIL-8 group was significantly higher than that in rENO group at 1, 2, 5 and 6 week s.v. respectively. Moreover, the ACH50 activity in rENO + ISA763 group was higher than that of rENO + rtIL-8 group at 1 ~ 8 week s.v. with significant difference of two groups from 5 to 8 week s.v.

#### Specific serum antibody response

**Figure 5:**
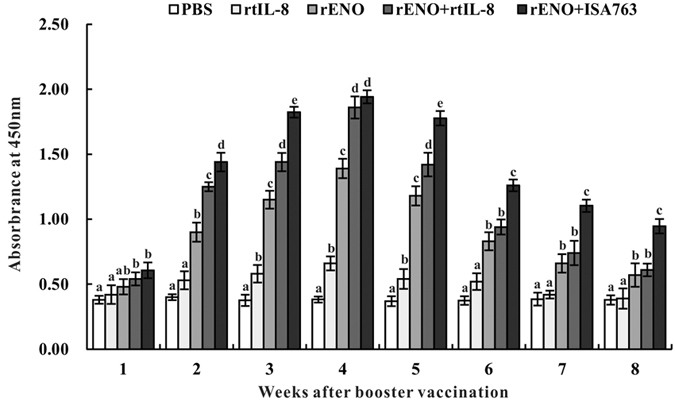
Specific serum antibody detection in vaccinated fish by ELISA. Channel catfish were vaccinated twice at 2-week intervals, with PBS, rtIL-8, rENO, rENO + rtIL-8 and rENO + ISA763. Sera were collected from the fish at 1 ~ 8 weeks s.v. Data are presented as means ± SD (n = 5). Different letters above a bar denote significant difference (*P* < 0.05).

The specific antibody titers in serum of vaccinated fish were detected continuously by ELISA from week 1 to 8 s.v. (Fig. 5). Generally, rENO, rENO + rtIL-8 and rENO + ISA763 stimulated significantly higher levels of antibodies than PBS and rtIL-8 from 2 to 7 week s.v. with the highest antibody level both peaking at 4-week s.v. The antibody levels of rENO + rtIL-8-vaccinated fish were remarkably higher than that in rENO-vaccinated fish from 2 to 5 week s.v., while rENO + ISA763 stimulated the production of antibody significantly than rENO + rtIL-8 at 2, 3, 5 ~ 8 week s.v. respectively. Moreover, the antibody levels induced by rtIL-8 alone was just higher than that in PBS-vaccinated fish at each of the examined time point except 3, 4 and 5 week s.v. (*P* < 0.05).

#### Immunoprotective efficacy against *S. iniae*

**Figure 6:**
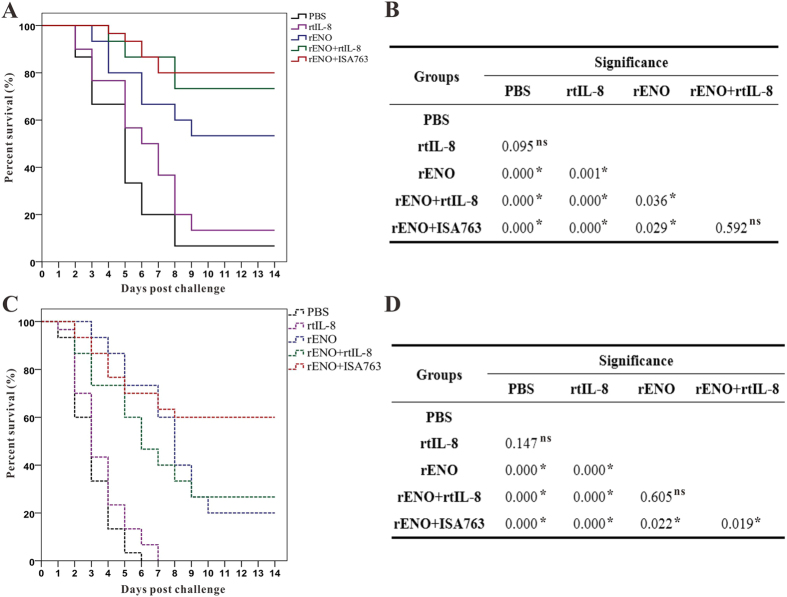
Percent survivals (Kaplan-Meier) of vaccinated fish during the challenges tests of 4 week s.v. (**A**) and 8 week s.v. (**C**). Differences among groups were tested using log-rank test shown in (**B**) (4 week s.v.) and (**D**) (8 week s.v.). “*”Denotes significant difference (*P* < 0.05), “ns” means not significant.

Fish vaccinated with PBS, rtIL-8, rENO, rENO + rtIL-8 and/or rENO + ISA763. exhibited accumulated survivals of 6.67%, 13.33%, 53.33%, 73.33% and 80.00% respectively (Fig. 6A) after challenging with the pathogenic *S. iniae* DGX07 at 4 week s.v. The survival of rENO + rtIL-8 and rENO + ISA763 was significant higher than rENO by log-rank test, while the difference of survival of two adjuvant groups was not significant (Fig. 6B). Furthermore, the immunoprotective efficacies, in terms of RPS, of rtIL-8, rENO, rENO + rtIL-8 and rENO + ISA763 were 7.14%, 50.00%, 71.43% and 78.57% respectively compared with PBS.

To examine the duration of protection, fish were also challenged at 8-week s.v., and the results suggested that the cumulative survivals of fish vaccinated with PBS, rtIL-8, rENO, rENO + rtIL-8 and rENO + ISA763 were 0%, 0%, 20.00%, 26.67% and 60% respectively (Fig. 6C), which correspond to an RPS of 20.00% for rENO group, 26.67% for rENO + rtIL-8 group and 60% of rENO + ISA763 group respectively. In addition, the results of log-rank test showed that the survival of rENO + ISA763 was significant higher than rENO + rtIL-8 and rENO, and the difference of survival of rENO + rtIL-8 and rENO was not significant (Fig. 6D). Furthermore, *S. iniae* DGX07 was the only type of bacterial strain recovered from the liver, and kidney of moribund fish during two challenge tests, suggesting that mortality was caused by *S. iniae* DGX07 infection.

#### Expression of immune-related genes

**Figure 7:**
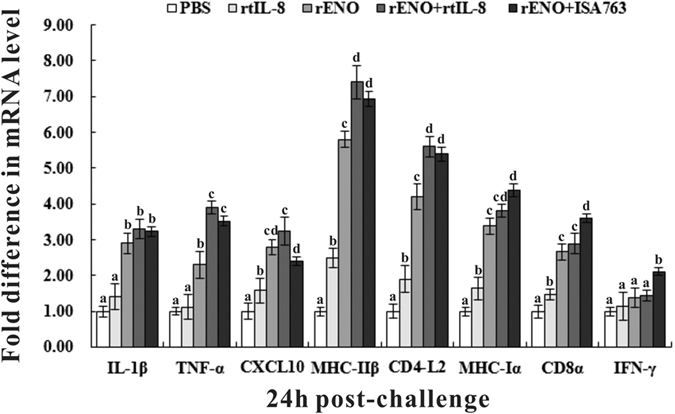
Expression of immune-related genes in vaccinated fish determined by qRT-PCR. Channel catfish were vaccinated twice at 2-week intervals, with PBS (control), rtIL-8, rENO, rENO + rtIL-8 and rENO + ISA763. Total RNA was extracted from the head-kidney at 24 h post-challenge of 4-week s.v. and used for qRT-PCR. For each gene, the mRNA level of the PBS-vaccinated fish was set as 1. Data are presented as means ± SD (n = 5). Different letters above a bar denote significant difference (*P* < 0.05).

To investigate the effect of vaccination on the expression of immune-related genes, qRT-PCR was carried out to analyze the expression of the genes encoding interleukin 1β (IL-1β), tumor necrosis factor-α (TNF-α), CXC Chemokine Ligand 10 (CXCL10), major histocompatibility complex class Iα (MHC Iα) and IIβ (MHC IIβ), CD4-L2, CD8α and interferon-γ (IFN-γ) with ACTB as an internal control. The results showed that except for IFN-γ, all other investigated genes were induced significantly in fish of rENO, rENO + rtIL-8 and rENO + ISA763 compared with the expression in PBS and rtIL-8 group, especially MHC II β and CD4-L2 with more than 4-fold expression (Fig. 7). The expression of CXCL10, MHC Iα, MHC IIβ, CD4-L2 and CD8α in rtIL-8-vaccinated fish were upregulated significantly compared with the control (PBS) and a slightly higher expression of IL-1β, TNF-α and IFN-γ. In addition, both rENO + rtIL-8 and rENO + ISA763 induced a significantly higher expression of TNF-α, MHC IIβ and CD4-L2, and stimulated a slightly higher expression of IL-1β, MHC Iα, CD8α and IFN-γ than that of rENO group. Moreover, vaccination with rENO + ISA763 upregulated the expression of CD8α and IFN-γ notably, stimulated a higher expression of MHC Iα and a lower expression of other investigated genes especially CXCL10 than that of rENO + rtIL-8.

## Discussion

An effective vaccine candidate usually consists of a strong immunogen (antigen) and a potent adjuvant (immunoenhancer) that can trigger early innate defense mechanisms to aid in the generation of robust and long-lasting immune responses^33^. However, most known effective adjuvants such as Freund’s complete adjuvant (FCA) and Freund’s incomplete adjuvant (FIA) are unsuitable for human and animal use owing to their toxicity and side effects^24^,^34^. It has been reported that co-vaccination of antigens with cytokines is a novel and effective strategy to enhance immunization and regulate cellular and humoral immune responses to promote protective immunity against pathogens. Hence, cytokines used as the potential adjuvants have been studied increasingly^22^,^23^,^24^,^35^,^36^,^37^. IL-8 as a CXC chemokine produced by numerous cell types, was known to recruit neutrophils to inflammatory sites. In mammals, it had been proved IL-8 was an important immune mediator for innate immunity involved in recruitment and activation of various leukocytes through modulating cytokine production^38^, and a potential immune adjuvant to promote cytokines immune response and increase the antibody response by co-administration with antigen^39^,^40^. However, there were few reports of the potential use of cytokines as an adjuvant in fish especially IL-8. Channel catfish as an important economic fish species, has served as a classical model for the study of comparative immunology. Research on the characterization and biological role of its cytokines should help in elucidation of innate immunity in catfish^32^.

In the present study, molecular cloning and recombination expression of mature IL-8 of channel catfish were performed, and rENO-vaccination alone and co-vaccination of rENO with rtIL-8 or commercial adjuvant Montanide™ ISA763 were conducted to evaluate the protective efficacy of rENO and the adjuvant efficacy of rtIL-8 against *S. iniae* in channel catfish. The results showed that the coding sequence of channel catfish IL-8 with 336 bp full-length encoded 111 a.a. belonging to the shorter alternatively spliced transcripts of IL-8 according to Chen *et al.*^32^, which existed 3 a.a. (9 bp) difference with the longer transcript. However, the 3-a.a. difference was located in the signal peptides based on sequence analysis (Fig. 1C), leading that both transcripts have the same mature peptide. Thus, we selected the truncate IL-8 (without signal peptides) to express the mature IL-8. In addition, it was interesting that ELR motifs lacking of leucine residue were found to be located immediately before the CXC motif of channel catfish IL-8 in our study, which was different from other fish IL-8 lacking of ELR motifs, indicating that the similarities among the fish IL-8 genes were relatively low (Fig. 2A,B) and the molecular evolution of channel catfish IL-8 occurred during the process of evolution and phylogeny to adapt to the special environment or living way.

Innate immunity as a fundamental defence mechanism of fish, plays an instructive role in the acquired immune response and homeostasis^25^,^41^. Lysozyme activity and alternative haemolytic complement activity (ACH50) are two important parameters to evaluate the innate immune response^41^. Both are involved in activating the complement system and play a critical role in the innate immune defence against bacterial and fungal pathogens^42^,^43^,^44^. In order to stimulate an optimal immune response, we boosted the immune system with the same method and dosage as first immunization at 2 week post-vaccination. The results of the present study showed that both rENO and rtIL-8 enhanced serum lysozyme activity and ACH50 activity, and resulted in stimulating the innate immune responses and activating the protective mechanisms. The lysozyme activity in rENO + rtIL-8-vaccinated fish increased significantly at 1 ~ 5 week s.v. and slightly increased at 6 ~ 8 week s.v. compared with that of rENO group, while lysozyme activity induced by rENO + ISA763 was remarkably higher than rENO + rtIL-8 at 5 ~ 8 week s.v. and the peak value in rENO + ISA763 group was also higher than that of rENO + rtIL-8 group. Similar trends were also observed on the ACH50 activity of rENO and rENO + rtIL-8 and two adjuvant groups, respectively.

Studies have suggested that production of antibodies in response to protein antigens is the basic mechanism of immunoprotection against *S. iniae* infection^7^. Antibody responses induced by subunit or DNA vaccines against *S. iniae* have been observed in various fish models^10^,^45^,^46^,^47^,^48^. In Japanese flounder vaccinated with recombinant Sip11 subunit vaccine against *S. iniae* SF1, serum specific antibodies were produced at 5 ~ 8 weeks post-vaccination and highest antibody titers were detected at the 6 week post-vaccination^46^. Similarly, specific antibody productions in turbot immunized with DNA vaccine pSia10 against *S. iniae* were detected at 4 ~ 7 weeks after immunization with the peak of antibody titers appearing at 5 and 6 weeks post-vaccination^45^. In the case of rENO, rENO + rtIL-8 and rENO + ISA763, we found that serum specific antibodies were both detected at 1 ~ 8 week s.v. and peaked at 4 week s.v., and the antibody levels of rENO + rtIL-8 group were remarkably higher than that of rENO group from 2 to 5 week s.v., while rENO + ISA763 induced significant higher specific antibody than rENO + rtIL-8 at 2, 3 and 5 ~ 8w s.v. respectively, which suggested that rtIL-8 enhanced the antibody levels induced by rENO but failed to hold this enhancement for longer time, and ISA763 not only increased the antibody levels but also maintained the increasement for long time compared with rtIL-8 adjuvant. Comparing with the above other vaccines against the same pathogen^45^,^46^, the time of antibody production and the peak in our study brought forward, mainly due to the difference of vaccines and the host fish, as well as different immune schedules.

Although specific antibodies are important for the immune response against *S. iniae* infection, they are not sufficient for complete protection which involves both humoral and cellular immunity^49^. Therefore, transcriptional analysis of immune-related genes were conducted by qRT-PCR in our study. The results showed that vaccination with rtIL-8, rENO, rENO + rtIL-8 and rENO + ISA763 exhibited comparable inductions in all the examined genes that related to inflammatory response (IL-1β, TNF-α and CXCL10), humoral immunity (MHC II β and CD4-L2) and cellular immunity (MHC Iα, CD8α and IFN-γ) compared with the control, especially MHC IIβ and CD4-L2 which participated in extracellular antigen presentation and the activation of T helper (Th) cells^50^, with more than 4-fold expression levels in rENO, rENO + rtIL-8 and rENO + ISA763 group. Compared with rENO, both rENO + rtIL-8 and rENO + ISA763 upregulated the expression of all genes with significantly higher expression of TNF-α, MHC IIβ and CD4-L2, which was, to a certain extent, in line with the protection efficacies exhibited by these vaccines. Besides, rENO + ISA763 upregulated a higher expression of MHC Iα, CD8α and IFN-γ, and a significantly lower expression of CXCL10 than that of rENO + rtIL-8. These results suggested that rENO induced a systemic immune response in channel catfish against *S. iniae* infection, and rtIL-8 activated inflammatory response and strengthened the immune response and ISA763 could promote innate immune reactions and cellular immunity^51^, which likely resulted in the higher level protection of rENO + rtIL-8 and rENO + ISA763.

RPS is not only one of the most visual indices to evaluate the immune effect in challenge test, but also a measure of vaccine efficacy against pathogen infection^52^. Our results suggested that rENO provided a moderate immune protection for channel catfish against *S. iniae* infection with the RPS of 50%, which was only half of the RPS (100%) of rENO in zebrafish against same pathogen^10^, chiefly because of the different fish species and infection dosages. Besides, the RPS of rENO alone in our study was also lower than the protection of other subunit vaccine against *S. iniae* including RPS (53.8%) of Sia10 in turbot^45^, RPS (69.7%) of Sip11 in Japanese flounder^46^ and RPS (66.9%) of mtsB in tilapia^47^, mostly due to the difference of vaccines, fish and immune schedules. However, adjuvants rtIL-8 and ISA763 enhanced the immune protection with RPS of 71.43% and 78.57% respectively at 4 week s.v., which were not significant each other but were significant higher than that of rENO alone. While rtIL-8 failed to maintain long period of immunity against *S. iniae* infection, only increased the RPS of rENO to 26.67% compared with rENO alone with RPS of 20.00% at 8 week s.v, and both were significant lower than rENO + ISA763 with RPS of 60.00%. In view of this, more effective way such as DNA vaccines like pSia10 with RPS of 73.9% in turbot against *S. iniae*^45^ and pSagF with RPS of 78% in Japanese flounder against *S. iniae*^48^ should be taken into account.

In conclusion, this study suggested that rtIL-8 hold promise for use as a potent immunopotentiator in subunit vaccines against *S. iniae* infections in channel catfish, while it is insufficient to extend the protective effects of rENO such as two month compared with the commercial adjuvant ISA763. Thus, our study supports the development of IL-8 as an effective adjuvant for subunit vaccine, enhancing the immune response and protection against bacterial infections in fish. However, further studies are required to understand the mechanisms of IL-8 used as an adjuvant and seek for more effective way to strengthen the adjuvanticity of IL-8 such as co-vaccination or fusion in DNA vaccines.

## Materials and Methods

### Bacterial strains, plasmids, reagents and growth conditions

*S. iniae* DGX07 is a pathogenic isolate from diseased channel catfish in China and stored at our laboratory^13^, it was cultured in Brain-Heart Infusion (BHI) medium at 37 °C. *E. coli* DH5α and *E. coli* BL21 (DE3) were used as the host strains for cloning and protein expression, respectively. Both were routinely grown in Luria-Bertani (LB) medium containing 100 μg/ml of ampicillin at 37 °C. The plasmids pMD19-T simple (Takara, Japan) and pET32a (+) (Merck, Germany) were used for T-A cloning and protein expression, respectively. Recombinant protein rENO was constructed, expressed and stored at our laboratory^20^. Montanide™ ISA763 A VG (Seppic, France) was selected as a commercial adjuvant for the experiment.

### Cloning of IL-8 complete coding regions

Total RNA was extracted from spleen of healthy channel catfish with RNAiso Plus Kit (TaKaRa, Dalian, China) and was reverse-transcribed into first-strand cDNA using PrimeScrip t™ RT reagent Kit with gDNA Eraser (Perfect Real Time) (TaKaRa) according to the manufacturer’s instructions. A channel catfish IL-8 complete coding sequence was cloned from the cDNA template using primers IL-8-F1/IL-8-R1 (Table 1), which were designed based on sequence published in GenBank (AY145142, corresponding protein GenBank: AAN60105) and by Primer Premier 5.0 software under default parameters. PCR amplification was performed under the following conditions: 1 cycle of 94 °C for 5 min, 30 cycles of 94 °C for 1 min, 55 °C for 30 s, and 72 °C for 30 s, followed by a final extension of 72 °C for 10 min. The PCR products were purified using the Agarose Gel DNA Extraction Kit (TaKaRa) and cloned into the pMD19-T vector, followed by transformation into *E. coli* DH5α. The positive recombinant clone was then selected using an Amp/IPTG/X-Gal agar plate. The recombinant plasmid was identified by PCR under the aforementioned conditions, digested with restriction enzymes *EcoR* I and *Xho* I, and fractionated on 1% agarose gels. DNA sequencing was also conducted by TaKaRa Bio Inc.

### Sequence and phylogenetic analyses

The amino acid (a.a.) sequence was derived from nucleotide sequence using the translate tool, the molecular weight and the isoelectric point (pI) of the peptide were calculated using ProtParam tool, the signal peptide was predicted with SignalP 4.1 program, both available on the ExPASy molecular biology server (http://www.expasy.org/tools)^53^. Multiple sequence alignments and the identities between each pair of the a.a. sequences were calculated by Clustal W method^54^ using MegAlign programe^55^, and the phylogenetic tree was constructed using the Neighbor-Joining (N-J) method in the MEGA 5.1 software^56^, with 1,000 bootstrap replicates.

### Cloning of truncate *IL-8* gene (*tIL-8*)

Based on the sequence analysis, the signal peptide was removed in order to obtain the mature IL-8 by prokaryotic expression with *E. coli* BL21 (DE3). The truncate IL-8 gene (tIL-8) was cloned from the cDNA template using degenerate primers IL-8-F2/IL-8-R2 (Table 1) which containing *EcoR* I and *Xho* I restriction enzyme sites. PCR amplification was performed as described above. The resultant amplicons were purified, cloned, transformed and sequenced, and the positive recombinant cloning plasmid named by T-tIL-8.

### Expression and purification of recombinant truncate IL-8 (rtIL-8)

The expression and purification of recombinant truncate IL-8 was conducted as described in our previous study^57^. Briefly, the plasmid T-tIL-8 was digested with EcoRI and XhoI and the resultant products were inserted into the EcoRI/XhoI-digested pET32a (+) vector to construct the recombinant expression plasmid, named as P-tIL-8. The plasmid was then transformed into *E. coli* BL21 and induced by adding 1.0 mM IPTG at 37 °C for 4 h. the cells were centrifuged at 8000× g for 10 min at 4 °C and suspended with sterile phosphate buffer saline (PBS), followed by ultrasonicating with an ultrasonic cell disrupter (JY92-IIDN, Ningbo Scientz, China) and examined by 12.5% SDS-PAGE. The inclusion bodies from the insoluble fractions were purified by Ni-NTA-Sefinose Column (Sangon Biotech, Shanghai, China) after dissolution in 8 M urea solutions and filtration with 0.22-μm filters. The refolding of the purified proteins was conducted by dialyzing gradiently from 6 M urea solutions to PBS at 4 °C, and then analyzed by 12.5% SDS-PAGE. The protein was quantified using the Bradford assay with bovine serum albumin (BSA) as standard and a NanoDrop spectrophotometer (Thermo Scientific) according to the manufacturer’s instructions. Purified proteins were named as rtIL-8 and stored at 20 °C.

### Western-blot analysis

The werstern-blot analysis of rtIL-8 was performed as previously described^57^. Briefly, the purified proteins were separated by 12.5% SDS-PAGE and transferred to a PVDF membrane at 150 V for 2 h. Non-specific binding sites of the membranes were blocked by incubating for 1 h at 37 °C in TBST (containing 3% BSA). Then the membrane was incubated with rabbit anti-6-Histidine antibody (Sangon Biotech, Shanghai, China) diluted 1:100 in TBST (containing 0.5% BSA) for 12 h at 4 °C. After washing 3 times with TBST, the membrane was incubated with goat-anti-rabbit IgG (H + L)-HRP (Sigma, St. Louis, MO, USA) diluted 1:5000 in TBST (containing 0.5% BSA) at 37 °C for 1 h. After washing off unbound secondary antibody, the specific antigen-bound antibody was visualized using DAB (Sigma) for 5 to 15 min, and terminated by rinsing with distilled water.

### Preparation of fish and vaccine

All animal experiments were approved by the Committee of the Ethics on Animal Care and Experiments at Sichuan Agricultural University. All experimental procedures were carried out in accordance with the approved guidelines.

Channel catfish (50.0 g ± 5.0 g) were purchased from a fish farm in Chengdu (Sichuan, China) and acclimatized in the laboratory for 2 weeks before experimental manipulation. The fish were fed a commercial diet daily, and water was partly replaced every day, maintaining a temperature of 28 °C ± 1 °C. Before experiments, fish were randomly sampled from blood, liver, kidney, and spleen, the examination of bacterial recovery indicated that no bacteria could be detected and agglutination test showed no reaction between the serum and *S. iniae* DGX07. Fish were anaesthetized with MS222 (Sigma, Beijing, China) prior to the experiments, which involved manipulations such as injections and serum collection. The purified protein rtIL-8 and recombinant protein rENO were diluted in PBS to 0.5 mg/ml and 1.0 mg/ml, respectively. To obtain rENO + rtIL-8, the recombinant protein rENO was mixed with an equal volumes of purified protein rtIL-8. To obtain rENO + ISA763, the recombinant protein rENO was mixed with adjuvant Montanide™ ISA763 A VG (Seppic, France) at a ratio of 3:7 by ultrasonic disruption (JY92-IIDN, Ningbo Scientz, China).

### Vaccination and bacterial challenge

Five hundred channel catfish were divided randomly into five groups (100 fish/group) and injected intraperitoneally (i.p.) with 0.2 ml of PBS (control group), rtIL-8, rENO, rENO + rtIL-8 and rENO + ISA763. The secondary vaccination were performed to obtain an optimal immune response with the same method and dosage at 2 week post-vaccination. At 4 and 8 weeks post secondary vaccination (s.v.), 30 fish from each group were randomly selected and challenged by i.p. injection with 0.2 ml of *S. iniae* DGX07 that resuspended in PBS to 6 × 10^7^ CFU/ml^57^. Mortality was monitored over a period of 14 days after the challenge, and dying fish were randomly selected for the examination of bacterial recovery from liver, kidney, and spleen. Relative percent of survival (RPS) was calculated according to the following formula: RPS = [1− (% mortality of vaccinated fish/% mortality of control fish)] × 100^58^. Serum samples of five fish in each group were collected for assessment of immune related indexes from 1 to 8 week s.v. and head-kidney of five fish were taken for qRT-PCR at 24 h post-challenge of 4 week s.v. All vaccination trials were repeated once.

### Detection of innate immune parameters

Serum lysozyme activity was measured by the turbidimetric method described by Hutchinson^59^. Briefly, 150 μL Micrococcus lysodeikticus with a concentration of 0.2 mg/mL (in 0.04 M PBS, pH = 6.2) was added to 15 μL sera in a 96-well U-bottom microtiter plate, quickly mixed by vortex, and the OD_450_ (UV absorption value at 450 nm) (A_0_) was assayed at 0.5 and 4.5 min after reaction, respectively. Each test was conducted in triplicate. One activity unit of lysozyme (U) was defined as the amount of serum lysozyme that caused a decrease in absorbancy of 0.001 per min at 450 nm.

Alternative haemolytic complement activity (ACH50) was determined following the method described by Sunyer and Tort^60^ based on haemolysis of rabbit red blood cells (RaRBC). The volume of serum yielding 50% haemolysis (ACH50) was determined and used to calculate the complement activity of the samples (value of ACH50 is in units per ml).

### Enzyme-linked immunosorbent assay (ELISA)

Specific antibody titers for rENO were determined by ELISA as described previously with some modification^57^. Briefly, the rENO was diluted to a concentration of 50 μg/mL in a carbonate buffer (pH = 9.6). Each well of the 96-well plate was covered with 100 μL diluted rENO overnight at 4 °C followed by washing with PBST (0.1% Tween-20 in PBS) and blocking with 3% BSA in PBST for 2 h at 37 °C. Serial 2-fold dilutions of sera were added to the wells in triplicate and subsequently incubated for 2 h at 37 °C. Rabbit anti-channel catfish IgM antibody (prepared in our laboratory) (1:2000) and goat-anti-rabbit IgG (H + L)-HRP (1:2000) were used as the secondary and tertiary antibodies, respectively. Color development was performed with the TMB kit (Tiangen, Beijing, China). The plates were read at 450 nm with a microplate reader (Bio-Rad, Hercules, USA).

### qRT-PCR analysis of the expression of immune-related genes

Head-kidney samples were taken from the fish (five in each group) at 24 h post-challenge of 4 week s.v. Total RNA extraction and cDNA synthesis were carried out as described above. qRT-PCR was performed with the SYBR^®^ Premix Ex Taq™ II (Tli RNaseH Plus) (TaKaRa) in an ABI StepOnePlus™ System (Applied Biosystems, USA) as described previously. Each assay was performed in triplicate with beta actin (ACTB) as an internal control. The primers used to amplify the immune-related genes are listed in Table 1. All data are given in terms of relative mRNA.

### Statistical analysis

All statistical analyses were performed with SPSS 19.0 software (SPSS Inc., USA). Mortality data from the challenge experiments were analyzed by the Kaplan-Meier methods and differences among groups were tested using log-rank test. The difference significance of other data were determined using a one-way analysis of variance (ANOVA). In all cases, the significance level was defined as *P* < 0.05 and the results were presented as means ± SD (standard deviation).

## Additional Information

**How to cite this article**: Wang, E. *et al.* Molecular cloning, expression and the adjuvant effects of interleukin-8 of channel catfish (*Ictalurus Punctatus*) against *Streptococcus iniae. Sci. Rep.* **6**, 29310; doi: 10.1038/srep29310 (2016).

## Figures and Tables

**Table 1:** Primers used for cloning, expression and qRT-PCR of genes in this paper.

Primers	Sequences (5′→3′)^a^	Target gene
IL-8-F1	CGGAATTCATGAAGGCTGCAACT (EcoRI)	IL-8
IL-8-R1	CCCTCGAGTCAGTTTTGCTGTTTG (XhoI)	
IL-8-F2	CGGAATTCGGAGAAGGAAAAGCAGAG (EcoRI)	tIL-8
IL-8-R2	CCCTCGAGTCAGTTTTGCTGTTTGATCT (XhoI)	
ACTB-F	CCCATCTATGAGGGTTATGCTCTG	ACTB
ACTB-R	GCTCGGTCAGGATCTTCATCAG	
IL-1β-F	GCCATGTTGCTAATGTTGTAATCG	IL-1β
IL-1β-R	TGTCTTGCAGGCTGTAACTCTTG	
TNF-α-F	CGCACAACAAACCAGACGAGAC	TNF-α
TNF-α-R	ACCACTGCATAGATACGCTCGAA	
CXCL10-F	ACAGGCCAGGACCAGTGTAAGG	CXCL10
CXCL10-R	CAAGTTGCACTCGCAGGATGAA	
MHC Iα-F	GGTATCATCGTTGGTGTAGCCG	MHC Iα
MHC Iα-R	GGACAGGTTTGAAGCCAGAGTT	
MHC IIβ-F	CGGGAAGGAGATTAAAGGAGGT	MHC IIβ
MHC IIβ-R	GTTTGGTGAAGCTGGCGTGT	
CD4-L2-F	GCAGGGCACGGATAGATGGA	CD4-L2
CD4-L2-R	TGGGTTCGCAGAGGCTGATAC	
CD8α-F	CCGACAGTGCCTACGACTAAAGC	CD8α
CD8α-R	CCAGCAGCCAAAGGAATGAAG	
IFN-γ-F	TGCACGAAGTGAAAGACCAAA	IFN-γ
IFN-γ-R	TTAAGGTCCAGCAGCTCAGTGA	

^a^Underlined nucleotides are restriction sites of the enzymes indicated in the brackets at the ends.
